# Advancements in melanoma immunotherapy: the emergence of Extracellular Vesicle Vaccines

**DOI:** 10.1038/s41420-024-02150-9

**Published:** 2024-08-23

**Authors:** Guijuan He, Yichuan Li, Yuyang Zeng, Yong Zhang, Qiong Jiang, Qi Zhang, Jinjin Zhu, Jun Gong

**Affiliations:** 1 Department of Plastic Surgery, Sir Run Run Shaw Hospital, Zhejiang University School of Medicine, Hangzhou, Zhejiang China; 2 Department of Dermatology, Tongji Hospital, Tongji Medical College, Huazhong University of Science and Technology, Wuhan, Hubei China; 3 Department of Pharmacy, Xianning Central Hospital, The First Affiliated Hospital of Hubei University of Science and Technology, Xianning, Hubei China; 4 Department of Plastic and Cosmetic Surgery, Tongji Hospital, Tongji Medical College, Huazhong University of Science and Technology, Wuhan, Hubei China; 5 Xianning Medical College, Hubei University of Science & Technology, Xianning, Hubei China; 6 Department of Dermatology, Union Hospital, Tongji Medical College, Huazhong University of Science and Technology, Wuhan, Hubei China; 7 Department of Biliary-Pancreatic Surgery, Affiliated Tongji Hospital, Tongji Medical College, Huazhong University of Science and Technology, Wuhan, Hubei China

**Keywords:** Drug delivery, Melanoma

## Abstract

Malignant melanoma represents a particularly aggressive type of skin cancer, originating from the pathological transformation of melanocytes. While conventional interventions such as surgical resection, chemotherapy, and radiation therapy are available, their non-specificity and collateral damage to normal cells has shifted the focus towards immunotherapy as a notable approach. Extracellular vesicles (EVs) are naturally occurring transporters, and are capable of delivering tumor-specific antigens and directly engaging in the immune response. Multiple types of EVs have emerged as promising platforms for melanoma vaccination. The effectiveness of EV-based melanoma vaccines manifests their ability to potentiate the immune response, particularly by activating dendritic cells (DCs) and CD8+ T lymphocytes, through engineering a synergy of antigen presentation and targeted delivery. Here, this review mainly focuses on the construction strategies for EV vaccines from various sources, their effects, and immunological mechanisms in treating melanoma, as well as the shortcomings and future perspectives in this field. These findings will provide novel insights into the innovative exploitation of EV-based vaccines for melanoma immune therapy.

## Facts

- Exosome vaccines, as an innovative therapeutic strategy, are emerging in the field of melanoma immunotherapy, with a unique mechanism that directly delivers tumor-specific antigens to immune cells, thereby triggering an immune response against melanoma.
- Exosome vaccines produced by melanoma cells contain abundant tumor-specific antigens, and after specialized processing, these exosomes can enhance the activity of dendritic cells and CD8+ T cells within the immune system, thus creating an effective antitumor immune environment in vivo.
- Exosome vaccines derived from non-tumor cells, such as dendritic cells and macrophages, have been shown in experimental studies to induce strong anti-tumor immune responses, promoting immune surveillance against melanoma by carrying tumor-associated antigens and immunostimulatory molecules.
- Although exosome vaccines hold great promise in melanoma treatment, their clinical application still faces several technical and methodological challenges, such as large-scale exosome extraction, standardized production, and ensuring their stability and bioactivity during long-term storage.

## Open questions

- Which source of extracellular vesicles (EVs), such as tumor cells, immune cells, or bacteria, proves to be the most effective in the development of melanoma vaccines?
- How can the production and purification techniques of EVs be optimized to achieve high yield and purity for clinical applications?
- Can EV-based vaccines achieve higher tumor specificity without causing damage to normal tissues?
- How should the optimal dosage and administration frequency of EV vaccines be determined in future clinical trials to ensure their safety and efficacy?

## Introduction

Melanoma is a highly aggressive form of skin cancer originating from the malignant transformation of melanocytes [1]. Melanoma can manifest in various anatomical locations including the skin, mucosal surfaces, the uveal tract of the eye, and the meninges [2]. Melanoma accounts for a minority of skin cancer cases with hypermalignancy [3]. Current therapeutic approaches for malignant melanoma encompass surgical resection, chemotherapy, radiotherapy, and the increasingly prominent field of immunotherapy. Surgery is typically reserved for early-stage melanoma and can effectively excise tumor tissues [4]. Chemotherapy and radiotherapy, although capable of destroying cancer cells, lack specificity and may inadvertently damage healthy cells, leading to adverse effects [5, 6].

Although advancements in early diagnostic capabilities have increased the likelihood of early detection and treatment, patients with advanced or metastatic melanoma still face poor prognosis [7]. Immunotherapy, as a mature therapeutic modality, minimizes damage to normal cells and is considered the mainstream option for treating advanced melanoma [8, 9]. This approach utilized the immune system to identify and attack melanoma cells. Immunotherapeutic strategies primarily include immune checkpoint inhibitors, cell therapies, and cancer vaccines [10, 11]. Immune checkpoint inhibitors, such as anti-PD-1 antibodies, can unblock the suppression of T cell activation by melanoma cells, thereby stimulating the anti-tumor immune response [12]. Unlike traditional vaccines against infectious diseases, the primary purpose of cancer vaccines is to treat rather than prevent disease [13]. Melanoma is a malignant tumor characterized by a high mutation burden, and various types of vaccines have been studied and developed, including dendritic cell vaccines, DNA vaccines, mRNA vaccines, peptide vaccines, and EVs-based vaccines [14]. These vaccines introduce tumor-specific antigens (TSAs) to provoke immune recognition and attack tumor cells, thereby achieving therapeutic effects. Due to its high tumor mutational burden (TMB), melanoma exhibits significant immunogenicity, capable of producing TSAs that are expressed exclusively in tumor cells, thus providing precise targets for cancer vaccine development [15–17].

Extracellular vesicles (EVs) are naturally occurring nanoscale particles secreted by nearly all cells [18, 19]. EVs carry a diverse array of biologically active molecules such as DNA, RNA, proteins, and lipids, including membrane proteins specific to themselves, characteristic nucleic acid sequences, and protein constituents that mirror the genetic characteristics of their cells of origin [20–22]. As essential facilitators of cellular interactions, EVs play a pivotal role in diseases such as cancer and autoimmune disorders [23, 24].

Notably, EVs have emerged as a novel vaccine platform, demonstrating immense potential in the treatment of melanoma. EVs can carry TSAs and directly stimulate the immune system [25, 26]. The uniqueness of EVs lies in their ability to direct origination from patient tumor cells, providing personalized and highly specific immune activation signals [27, 28]. Researchers identify unique mutational profiles in tumors through Next-Generation Sequencing (NGS) and develop personalized EV vaccines targeting these mutations [29, 30]. These vaccines not only enhance the precision and specificity of treatment but also augment anti-cancer efficacy, offering novel methods to patients with advanced melanoma [31]. Therefore, this review aims to provide a comprehensive overview of the construction strategies for EV vaccines from various sources, their effects, and immunological mechanisms in the treatment of melanoma, while also presenting reflections on the shortcomings and future perspectives in this field. These insights will pave the way for innovative therapeutic strategies in the development of EV-based cancer vaccines.

## EVs potential as tumor vaccines

**Fig. 1:**
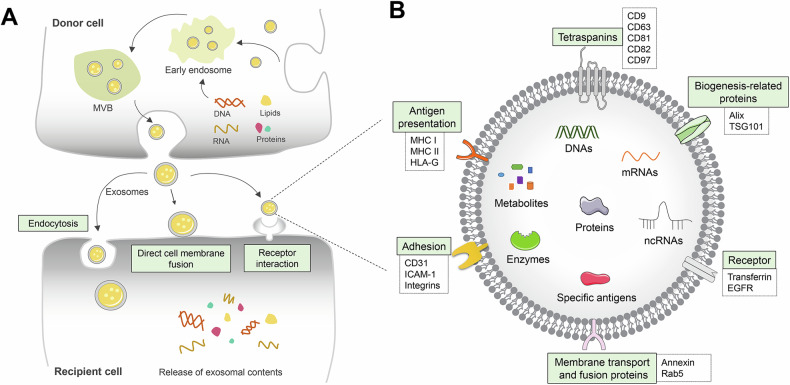
Exosome biogenesis and markers. **A** Extracellular vesicles (EVs), primarily composed of exosomes, undergo a complex biogenesis process involving early endosomes, multivesicular bodies (MVBs), and the formation of exosomes. Once separated from the parent cell into the extracellular space, exosomes can enter recipient cells through three primary mechanisms: endocytosis, direct cell membrane fusion, and receptor interaction. Subsequently, upon internalization by the recipient cells, exosomes can release their cargo of bioactive components, triggering a cascade of signaling reactions and influencing the biological functions of the recipient cells. **B** Exosomes contain numerous characteristic cell membrane molecules and cargo molecules. These characteristic membrane molecules are associated with various biological behaviors, including antigen presentation, tetraspanins, biogenesis-related proteins, receptors, membrane transport and fusion proteins, and adhesion. The cargo molecules carried by exosomes include DNAs, mRNAs, ncRNAs, proteins, metabolites, enzymes, and specific antigens.

EVs are lipid bilayer vesicles released into the extracellular environment by almost all types of cells via autocrine or paracrine mechanisms [32]. Based on their diameters and biogenesis, EVs are classified into three main subtypes: exosomes, microvesicles (MVs), and apoptotic bodies (ABs) [33]. Exosomes typically range from 30 to 150 nanometers in diameter [34]. The biogenesis of exosomes can be divided into four critical stages, beginning with the deformation and invagination of the plasma membrane to form early endosomes [35, 36]. As early endosomes mature, early endosomes develop into multivesicular bodies (MVBs) [37]. Then, MVBs undergo biogenesis by germinating and budding, producing intraluminal vesicles (ILVs) surrounded by endosomes [38]. Finally, MVBs fuse with the plasma membrane and release exosomes [39]. MVs ranging from 150 to 1,000 nanometers, are formed through the outward budding and fission of cholesterol-rich cell membranes [40, 41]. ABs range from 500 to 2000 nanometers and are formed during apoptosis through cysteine asparaginase-mediated rupture of cellular structures and blistering of cellular membranes [42]. Once released outside, EVs can enter target cells via membrane fusion, endocytosis, or ligand-receptor interactions [43]. By delivering nucleic acids, proteins, lipids and multimolecular complexes, and other biologically active components, EVs mediate intercellular communication and intercellular substance exchange, and are involved in numerous biological functions, including innate and adaptive immunity, immunomodulation, and the defense mechanisms against tumors [44, 45] **(**Fig. 1**)**.

One hallmark of cancer is the ability of tumor cells to evade immune surveillance by suppressing immune responses [46]. The formation, progression, and metastasis of cancer closely depend on the complex interactions between tumor cells with surrounding cells within the tumor microenvironment (TME) [47]. Tumor-derived EVs (T-EVs) are critical for tumor remodeling with dual functions of immune evasion and immunogenicity. Regarding immune evasion, cancer-derived EVs impact a broad range of immune cells, including T cells, NK cells, myeloid-derived suppressor cells (MDSCs), tumor-associated macrophages (TAMs), and neutrophils [48, 49]. These T-EVs directly inhibit the activity and functions of these cells, and modulate the immune microenvironment, thereby facilitating tumor growth and dissemination [50]. Additionally, melanomas can promote sustained tumor growth by releasing EVs carrying immune-suppressive molecules such as PD-L1 on their surface, leading to the inactivation of CD8+ T cells [51].

More importantly, the feature of immunogenicity possessed by T-EVs is essential for tumor vaccine development [52, 53]. T-EVs could effectively activate the anti-tumor immune response by carrying tumor-associated antigens (TAAs), damage-associated molecular patterns (DAMPs), and other immunomodulatory molecules [54, 55]. The TAAs contained within T-EVs directly stimulate immune cells [56]. After these EVs are phagocytosed by DCs, the internal TAAs are processed and presented to T cells, subsequently initiating a specific anti-tumor immune response. In addition, T-EVs can enhance the recognition of tumor cells by cytotoxic T lymphocytes (CTLs) and increase NK cell-mediated tumor cell-killing activity by boosting IFN-γ secretion. Furthermore, double-stranded DNA within T-EVs functions as DAMPs, activating the cGAS/STING pathway to enhance the expression of Type I interferons, thereby boosting the anti-tumor immune response [57, 58]. Moreover, radiotherapy or chemotherapy can alter the composition of T-EVs, such as increasing the DNA content, which can activate the STING-dependent pathway in DCs, thereby enhancing the immune response against the tumor [59, 60].

In addition to T-EVs, EVs from other sources have also been found to activate or modulate the immune system, providing a broad range of strategies for the development of cancer vaccines [61]. Furthermore, through various engineering modifications, these EVs can be endowed with the ability to carry specific TAAs and immune-activating factors, thereby enhancing the effectiveness of adaptive immune response against tumors [62, 63].

## EVs as vaccines for treating melanoma

### Melanoma cell-derived EVs as melanoma vaccines

T-EVs are a double-edged sword in cancer progression, with the potential to promote tumor growth and immune suppression, yet they can also act as mediators to activate immune responses against cancer [64]. Melanoma cell-derived EVs (M-EVs) inherently contain a range of TAAs, capable of inducing specific anti-tumor immune responses, making them ideal vaccine candidates [65].

EVs derived from B16-F10 melanoma cells (B16-EV) could carry tumor antigens, facilitate maturation, activate CD8+ T cells, and increase the IFN-γ level, thus inhibiting the growth and metastasis of melanoma in mice [66]. Ma et al. discovered that DCs internalizing microparticles derived from B16-OVA melanoma cells (OVA-MPs) triggered reactive oxygen species (ROS) response catalyzed by NOX2 [67]. This response elevated lysosomal pH and facilitated the production of major histocompatibility complex (MHC)-tumor antigen peptide complexes, while also stimulating the release of Ca2+ and the activation of transcription factor EB (TFEB). Such mechanisms enhance the expression of co-stimulatory molecules CD80 and CD86, ultimately boosting the activation of tumor-lytic CD8+ T cells.

These findings position tumor cell-derived EVs as promising candidates for cell-free tumor vaccines, emphasizing their potential in prophylactic anti-tumor immunization strategies. Furthermore, EVs can be engineered through pretreatment, genetic modification, or chemical modification to express specific antigens or immunomodulatory factors, accordingly enhancing their immunostimulatory effects and offering novel personalized vaccine approaches [68, 69].

#### Effects of radiation and chemotherapy on melanoma cell-derived exosomes and immune therapy

Following drug and radiation treatments, melanoma cells undergo a series of cascading genetic and functional alterations, which are subsequently reflected in the composition of EVs [70, 71]. This pretreatment paradigm could potentially amplify the antitumor effects of EVs, suggesting a novel approach to enhance the efficacy of melanoma therapies. For example, Oxaliplatin is a chemotherapeutic agent that prevents the replication and proliferation of cancer cells by reducing DNA synthesis [72, 73]. Stritzke et al. found that oxaliplatin could induce immunogenic cell death (ICD) in B16-OVA melanoma cells, leading them to release EVs carrying antigens and DAMP [74]. These EVs could activate CD8+ T cells in the spleen to produce IFN-γ, which triggered a tumor-specific immune response and enhanced the immunosurveillance and therapeutic effects of melanoma. Thus, oxaliplatin not only acted through a straight killing mechanism, but also indirectly enhanced the recognition and attack of melanoma by the immune system, demonstrating its potential dual role in melanoma immunotherapy.

Gamma radiotherapy promoted T cell recognition of irradiated tumor cells by enhancing MHC class I molecule-mediated antigen presentation and activating the mTOR pathway, and also facilitated the production of more immunogenic exosomes by irradiated cells [75, 76]. Kim et al. found that gamma-irradiated melanoma cancer cell-derived exosome significantly promoted DC maturation, enhanced the expression of surface molecules, the release of pro-inflammatory cytokines, antigen-presenting capacity, and reduced endocytosis, which resulted in effective activation of T cells, particularly Th1 and IFN-γ-producing CD8+ T cells, ultimately significantly decreasing the tumor growth in melanoma mice [77]. In contrast, non-irradiated melanoma cancer cell-derived exosome resulted in a semi-mature state of DCs, inhibiting T cell proliferation and IFN-γ production while increasing IL-10-producing CD4+ T cells, demonstrating weaker anti-tumor effects. This implied that gamma irradiation boosted the immune activation efficacy of exosome vaccines, for eliciting intense immunogenicity and tumor-specific T-cell responses. In summary, preconditioned melanoma EVs by radiotherapy and chemotherapy can significantly improve their effectiveness in melanoma vaccine immunotherapy.

#### Gene-modified melanoma cell-derived EVs

By employing gene editing techniques, it is possible to engineer tumor cells to overexpress TSAs or specific RNA/protein molecules in their secreted EVs [78, 79]. These modification strategies could effectively potentiate the anti-tumor immune response, presenting individually effective cancer immunotherapies [80–82]. Koyama et al. engineered B16 melanoma cells via transfection to express the Mycobacterium tuberculosis (MTB) antigen ESAT-6, thus successfully constructing exosomes carrying both tumor-specific and ESAT-6 antigens [83]. These exosomes boosted robust cellular immune responses against both B16 cells and ESAT-6 in mice, significantly inhibiting tumor growth upon direct intratumoral injection. Furthermore, Semionatto et al. engineered B16-F10 melanoma cells to express TNFSF ligands 4-1BBL and OX40L respectively, resulting in EVs formation that activated the proliferation of CD4+ T cells and inhibited the expression of Treg cells. These complex EVs strengthened the anti-tumor response, providing the possibility of developing anti-tumor vaccines for EVs based on TNFSF ligands [84].

Large tumor suppressors 1 and 2 (LATS1/2), as a key component of the Hippo signaling pathway, prevent tumor development by maintaining tissue homeostasis and inhibiting cellular hyperproliferation [85]. However, LATS1/2 deletion has the opposite effect and enhances the immunogenicity of tumor cells. In melanoma mouse models, Moroishi et al. revealed that using LATS1/2-null B16-OVA melanoma cells as a vaccine, significantly enhanced the immune response against melanoma [86]. These LATS1/2-null tumor cells induced type I interferon production by releasing EVs and activating the TLRs-MYD88/TRIF pathway, thereby potentiating tumor immunogenicity and effectively suppressing melanoma growth. In summary, functional potentiation of tumor cell-derived EVs by altering the properties of the tumor cells through gene editing techniques can be used as a strategy to enhance tumor vaccine efficacy.

#### Melanoma cell-derived modified EVs

In addition to genetically engineering melanoma cells, the further modification of EVs derived from melanoma cells is also of paramount importance. It will be more effective in EV interaction with DCs, by incorporating specific molecular markers and immune activators into these EVs. The modified EVs essentially function as specialized vehicles, delivering their cargoes directly to DCs to arouse robust immune response. This method is a highly specialized, targeted treatment based on directional modification of EVs [87].

DC-specific intercellular adhesion molecule-3-grabbing non-integrin (DC-SIGN) is a transmembrane C-type lectin mainly expressed on DCs that facilitate antigen capture and delivery to MHC pathways, exhibiting potential in activating T cells [88]. Horrevorts et al. formulated apoptotic tumor cell-derived EVs (ApoEVs) as a unique tumor-specific antigen carrier [89]. These ApoEVs were modified to express a high-mannose structure to target DC-SIGN on the monocyte-derived DCs to enhance vesicle internalization, thus further initiating and activating tumor-specific CD8+ T cells. ApoEVs could carry tumor-specific neoantigens and other TAAs could be a personalized and broad-spectrum immunotherapy option for patients.

CpG-DNA, an immunostimulant, can induce maturation and activation of DCs by targeting TLR9, thereby activating specific immune responses [90]. Matsumoto et al. created a self-assembled micron-sized CpG-DNA-modified tumor cell-derived sEV superstructure (CpG-sEV assembly) [91]. CpG-sEV assembly treatment promoted TNF-α and IL-6 release from DC2.4 cells. In addition, the CpG-sEV assembly significantly prolonged the residence time, and elicited elevated cytokine and chemokine levels, such as IL-6, IL12-p40, and CXCL5, along with increased Th1-associated IgG2a antibody specific to B16BL6 melanoma in vivo. Thus, CpG-sEV assembly effectively fostered melanoma-specific immune response, posing the potential as a prospective melanoma vaccine.

In addition, their team also prepared streptavidin-lactamyxin (SAV-LA)-modified exosomes (SAV-exo) successfully by transfecting B16BL6 cells with SAV-LA fusion proteins and subsequently combined them with biotinylated CpG-DNA to prepare CpG-DNA-modified exosomes (CpG-SAV-exo) [92]. CpG-SAV-exo could efficiently stimulate DC2.4 cells, promote the release of TNF-α, IL-6, and IL-12p40, and reinforce tumor antigen presentation, which induced a potent Th1-type humoral immune response and remarkable CTL activity, thereby demonstrating a robust anti-tumor effect in B16BL6 melanoma-bearing mice. In addition, CpG-SAV-exo immunization significantly prolonged the survival and inhibited tumor metastasis without inducing angiogenesis in tumor tissues.

For enhancing tumor antigen presentation, they further conjugated SAV-exo with fusogenic GALA peptide to produce GALA-modified exosomes (GALA-exo) [93]. GALA-exo possessed membrane cleavage activity in an acidic environment, which enhanced the cargo delivery capability in the cytoplasm of DC2.4 cells, for strengthening the tumor antigen presentation ability of MHC class I molecules. This effect ultimately enhanced the efficiency of activating specific T-cell responses in DC2.4 cells.

Surface modification of EVs by chemical approach enables the lipid or protein constructs of EVs to covalently couple with different junction motifs, ultimately conferring specific functions to the EVs [94]. Bhatta et al. introduce a novel metabolic tagging technology that embeds unique chemical tags, such as azido groups, onto the surface of EVs, enabling their modification via click chemistry for enhanced tracking and targeted delivery [95]. This approach significantly enhanced DC activation and CD8+ T cell responses when applied to tumor-derived EV vaccines without increasing toxicity, demonstrating substantial efficacy against B16-F10 melanoma, respectively. These findings offer a universal strategy for producing chemically modified EVs, opening new avenues for the development of highly effective EV-based vaccines and therapeutics.

**Fig. 2:**
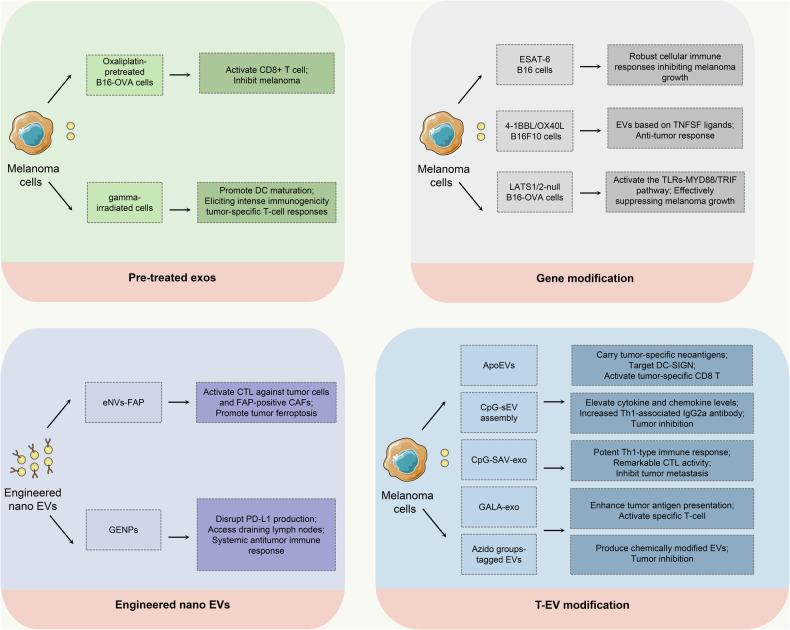
Strategies for melanoma vaccine construction based on melanoma-derived EVs. EV vaccines in melanoma immunotherapy mainly consist of pretreatment of melanoma cells (Oxaliplatin, gamma irradiation), and Gene modification (ESAT-6, 4-1BBL/OX40L, LATS1/2-null B16-OVA) before obtaining EVs, and direct modification of T-EVs (ApoEVs, CpG-sEV, SAV-LA, SAV-exo, GALA peptide, Azido groups), and engineered nano EVs (eNVs-FAP, GENPs). These obtained vaccines based on melanoma-derived EVs possessed prominent DC maturation promotion, CTL effects, and tumor-killing effects.

In summary, modified EVs obtained by genetic engineering, chemical modification, and other methods show potential as tumor vaccines and antigen delivery systems, providing innovative avenues for cancer therapy (Fig. 2).

#### Engineered nano EVs for melanoma immune therapy

Engineered nano EVs can efficiently deliver TSAs and leverage their inherent biocompatibility and intercellular communication capabilities for effective targeting of TME [96]. In addition, engineered nano EVs can be designed to contain various immunomodulatory molecules, thereby enhancing the multifunctionality and efficacy of the cancer vaccine [97].

Cancer-associated fibroblasts (CAFs), as key cellular components of the TME, can induce the proliferation, invasion, and stemness of cancer cells, promote epithelial-mesenchymal transition (EMT), and suppress immune responses [98, 99]. Fibroblast activation protein-α (FAP), is highly expressed in CAFs within various tumor tissues, and is considered a crucial molecule engaging in TME remodeling and tumor evolution [100]. Hu et al. prepared a nanovaccine based on genetically engineered tumor cell exosomes of FAP (eNVs-FAP) targeting both tumor parenchyma and CAFs [101]. In the melanoma xenograft model, the eNVs-FAP vaccine effectively inhibited tumor growth by promoting the maturation of DCs, activating specific CTL responses against tumor cells and FAP-positive CAFs, and reducing the proportion of M2-like TAMs, MDSCs, and regulatory T cells (Tregs) to remodel the TME. In addition, the eNVs-FAP vaccine further enhanced the immune response against tumors by stimulating the release of γ-interferon from CTL and promoting the depletion of FAP-positive CAFs to promote tumor ferroptosis. Moreover, the combination of eNVs-FAP with the ferroptosis agonist RSL3 enhanced the ferroptosis response by increasing LPO levels in tumor tissues, resulting in a synergistic anti-tumor effect.

In metastatic melanoma, tumor cells express PD-L1 proteins on the surface and release PD-L1-bearing exosomes, which circulate throughout the body, impairing the function of T cells in the draining LNs (DLNs) and spleen, leading to systemic immunosuppression [102]. Ye et al. developed a novel type of nanoparticles, Golgi apparatus-Pd-l1^−^^/−^exosome hybrid membrane coated nanoparticles (GENPs), which were capable of interfering with PD-L1 synthesis and PD-L1-bearing exosome secretion within the Golgi of tumor cells, thus possessing the potential to reverse the immunosuppressive TME [103]. GENPs were able to mimic exosomes into DLNs and induce T cell activation through vaccine-like effects, strongly promoting systemic immune responses. In mice models with incomplete metastatic melanoma resection, the combination of GENPs with anti-PD-L1 treatment in the sprayable in situ hydrogel restored the immune cell activity in spleen and DLNs, inhibited local residual tumor growth and metastasis, as well as suppressed tumor recurrence and significantly extended survival. This novel nanovaccine treatment offered an effective approach to the postoperative treatment of melanoma, for overcoming immunosuppressive TME and preventing tumor recurrence (Table 1).

**Table 1:** Melanoma-derived EVs for constructing melanoma vaccines.

Name	Source of exosomes	EV Vaccine Antigens	Main function	Ref.
Oxaliplatin-pretreated B16-OVA melanoma cell-derived EVs	B16-OVA melanoma cells	B16-OVA	Activating CD8+ T cells in the spleen to produce IFN-γ	[74]
Gamma-irradiated melanoma cancer cell-derived exosomes	B16/BL6 melanoma cells	HMGB1	Promoting DC maturation, activating Th1 and IFN-γ-producing CD8+ T cells, and ultimately significantly reducing tumor growth in melanoma-bearing mice	[77]
ESAT-6-modified B16 melanoma cell-derived exosomes	B16 melanoma cells	ESAT-6 antigens	Inhibiting tumor growth upon direct intratumoral injection	[83]
4-1BBL- and OX40L-engineered B16-F10 melanoma cell-derived EVs	B16-F10 melanoma cells	TNFSF ligands 4-1BBL and OX40L	Activating the proliferation of CD4+ T cells and inhibiting the expression of Treg cells	[84]
LATS1/2-null B16-OVA melanoma cell-derived EVs	LATS1/2-null B16-OVA melanoma cells	B16-OVA	Inducing type I interferon production and activating the TLRs-MYD88/TRIF pathway, thereby potentiating tumor immunogenicity and effectively suppressing melanoma growth	[86]
Glycan-modified apoptotic melanoma cell-derived EVs	The human HLA-A2 negative melanoma cells	High-mannose structure binding with DC-SIGN	Targeting DC-SIGN on the monocyte-derived DCs and activating tumor-specific CD8+ T cells	[89]
CpG-DNA-modified tumor cell-derived sEVs(CpG-sEV assembly)	B16BL6 melanoma cells	CpG-DNA, SAV-LA, Gaussia luciferase-LA	Eliciting elevated cytokine and chemokine levels, along with increasing Th1-associated IgG2a antibody specific to B16BL6 melanoma	[91]
CpG-DNA-modified SAV-LA-expressing exosomes (CpG-SAV-exo)	B16BL6 melanoma cells	CpG-DNA, SAV-LA	Stimulating DC2.4, inducing a potent Th1-type humoral immune response and remarkable CTL activity, and inhibiting tumor metastasis	[92]
GALA-modified exosomes (GALA-exo)	B16BL6 melanoma cells	GALA peptide, SAV-LA	Enhancing the cargo delivery capability in the cytoplasm of DC2.4 cells, strengthening the tumor antigen presentation ability of MHC class I molecules	[93]
Azido groups-tagged EVs	B16-F10 melanoma cells	Azido groups, TLR9 agonists (DBCO-modified CpG)	Enhancing DC activation and CD8+ T cell responses	[95]
eNVs-FAP	Engineered B16-F10 tumor cells	FAP	Inhibiting tumor growth by promoting the maturation of DCs, activating specific CTL responses against tumor cells and FAP-positive CAFs, and stimulating the release of γ-interferon from CTL and promoting the depletion of FAP-positive CAFs to promote tumor ferroptosis. Enhancing the ferroptosis response when combined with the ferroptosis agonist RSL3	[101]
GENPs	B16-F10 Golgi Apparatus membrane, B16-F10 Pd-l1^−/−^exos membrane	GEM, PLGA-RA	Restoring the immune cell activity in spleen and DLNs, inhibiting local residual tumor growth and metastasis, and suppressing tumor recurrence when combined with anti-PD-L1 treatment in the sprayable in situ hydrogel	[103]

### Dendritic cell-derived EVs as melanoma vaccines

DCs are sentinels of the immune system and perform an indispensable role in tumor immunotherapy, serving as the key initial cells in triggering anti-tumor responses [104]. As antigen-presenting cells, DCs process and present antigens via MHC-like molecules to CD4+ and CD8+ T cells, thereby initiating a robust immune response against tumor cells [105]. In tumor immunotherapy, the significance of DC-derived exosomes (Dex) in the antigen-presentation cascade is increasingly recognized [106]. Dex could ferry and convey antigenic information, thereby substantially augmenting T-cell activation [107]. Moreover, Dex has successfully addressed the challenges of cost-effectiveness and stability associated with traditional DC vaccines, offering significant advantages and opening new avenues in cancer treatment [108].

Toll-like receptors (TLR) agonists activate the maturation and function of DCs by mimicking danger signals during infection, leading to more effective T cell activation and tumor immune response [109]. Damo et al. produced the Dex vaccine loaded with antigens and matured using TLR agonists like poly (I:C) [110]. These Dex elicited T-cell responses and recruited cytotoxic CD8+ T cells, NK, and NK-T cells to the tumor site, demonstrating intense anti-tumor effects in the B16-F10 melanoma model. These findings underscore the potential of optimized Dex formulations in cancer immunotherapy, highlighting poly (I:C) as an effective adjuvant for enhancing vaccine efficacy.

CSF-1R is a crucial tyrosine kinase receptor intimately associated with the regulation of the TME and the recruitment of TAMs [111]. CSF-1R plays a pivotal role in controlling the development and function of immune cells, representing a potential target for cancer therapy. Exosomes isolated from tumor antigen-stimulated mature DCs in the presence of a maturation cocktail and tumor antigen (mDex^TA^) exhibit high levels of MHCs and co-stimulatory molecules expression and activate primitive DCs and T cells more efficiently in vitro, thereby exerting potent anti-tumor responses. Barnwal et al. discovered that the combination therapy of mDex^TA^ and CSF-1R inhibitor PLX-3397 effectively strengthened the infiltration of CD8+ T cells in TME, facilitating a shift from the Th1/Th2 balance to Th1 predominance, and decreasing immune-suppressor cells, such as TAMs and MDSCs in the B16-F10 murine melanoma model, thereby inhibiting tumor growth and enhancing survival rates [112]. In addition, this combination therapy increased CD8+ T cell populations in the spleen and lymph nodes (LNs), thus promoting a systemic anti-tumor immune response. This combination strategy emerges as an innovative and efficacious therapeutic option for the treatment of melanoma.

Dex contains all the necessary immunostimulatory components and exhibits excellent performance as an effective cell-free alternative vaccine delivery regimen [113]. However, exosome-based vaccines containing TAAs may induce immune tolerance and pose an autoimmune risk, as TAAs are also expressed in normal tissues, which may limit their therapeutic efficacy. To overcome this, combining neoantigens present only in tumor cells into a personalized vaccine formulation can trigger a targeted immune response. Li et al. designed a vaccine platform that integrates a Dex carrier with a patient-specific neoantigen [114]. The ovalbumin-loaded exosome (Exo-OVA) exhibited excellent lymphoid-directed migration properties and long-lasting antigen retention, thereby stimulating strong immune responses via antigen-specific broad-spectrum T cells and B cells, while maintaining a high degree of biosafety and compatibility. In addition, they loaded the newly identified novel epitopes M27 and M30 mutated in B16-F10 melanoma into exosomes (Exo-M27/M30). Exo-M27/M30 effectively inhibited tumor growth, extended survival periods, improved survival rates, and delayed tumor progression, while also eradicating pulmonary metastasis in therapeutic, prophylactic, and metastatic B16-F10 melanoma models respectively. This strategy of combining neoantigens into vaccines contributes to effective tumor control while minimizing the risk of autoimmunity, and represents a strategic advance in vaccine-mediated cancer immunotherapy.

### Macrophage-derived EVs as melanoma vaccines

Macrophages, as members of the remarkably heterogeneous monocyte-macrophage lineage spectrum, are significantly influenced by the surrounding microenvironment [115]. Macrophages can be classified into pro-inflammatory M1 and anti-inflammatory M2 types based on their activation state [116]. M1-derived exosomes (M1 exosomes) are enriched with immune-activating molecules that promote immune activation in the local TME, thereby enhancing the ability of the immune system to recognize and attack melanoma cells [117]. Xu et al. developed a lipid calcium phosphate (LCP)-based vaccine platform that could successfully deliver the melanoma-specific antigen TRP2 to mice in the B16-F10 melanoma model to stimulate a powerful cytotoxic T cell-mediated immune response [118]. To enhance the immunogenicity of the vaccine, their team then explored the application of M1 exosomes as an endogenous immune booster. M1 exosomes efficiently migrated to LNs and activated macrophages and DCs, thereby promoting the release of Th1-type cytokines [119]. Compared to conventional CpG oligonucleotide adjuvants, M1 exosomes exhibited superior immune-enhancing effects when combined with LCP vaccines, as reflected by significant inhibition of tumor growth, increased apoptosis of cancer cells, and enhanced immune cell infiltration.

Studies have demonstrated that exosome therapies based on cancer cells and their components can specifically target tumors, exhibiting distinct homing capabilities [120]. Wang et al. imported nuclei isolated from B16 mouse melanoma cells introduced into activated M1-like macrophages and then prepared biologically reprogrammed chimeric exosomes (aMT-exos) [121]. In B16 tumor-bearing mice, aMT-exos enabled the dual accumulation of aMT-exos in LNs and tumor tissues, and aMT-exos strongly triggered cellular and humoral immune responses in LNs through both traditional antigen presentation and direct exosome interaction manner. In addition, aMT-exos increased the immunoreactive component of TME and decreased the immunosuppressive component, ultimately causing tumor regression and prolonging survival. Furthermore, when combined with a-PD1 therapy, aMT-exos extended survival in mouse models of metastatic and post-surgical tumor recurrence, demonstrating synergistic effects with existing immunotherapeutic treatments and great promise in clinical tumor immunotherapy.

### iPSCs-derived EVs as melanoma vaccines

Induced pluripotent stem cells (iPSCs) are stem cells obtained by reprogramming into somatic cells with the potential to differentiate into any cell type. iPSCs have emerged as a multifunctional platform for tumor immunotherapy, enabling the generation of DC cells and patient-specific CTLs, through gene editing and guided differentiation techniques [122]. Moreover, iPSCs are capable of generating exosomes (iPSC-EXOs) containing tumor antigens, which directly participate in immunomodulatory processes. Combining iPSC-EXOs with DCs to create personalized vaccines offers a novel strategy to elicit robust tumor-specific immune responses, potentially transforming the treatment paradigm for relapsed or refractory cancers. Wang et al. showed that human iPSC-EXOs and murine iPSC-EXOs could function as valid adjuvants for DC vaccines, facilitating T-cell activation and triggering a robust immune response against melanoma cells [123]. In the mouse melanoma model, the DCs pulsed with iPSC-EXOs (DC + EXO) vaccine notably inhibited tumor growth and lung metastasis, while inducing durable immune memory to prevent tumor recurrence, accompanied by possessing excellent biocompatibility. The research highlighted the anti-tumor effects of the DC + EXO vaccine and also emphasized the prospective application for personalized iPSC-based vaccine development.

### hAEC-derived EVs in melanoma vaccines

Human amniotic epithelial cells (hAEC) are derived from the placenta and have pluripotency, immunomodulatory properties, and low immunogenicity [124]. hAECs demonstrate a unique dual functionality, contributing to both tissue repair and acting as potential anti-tumor vaccines [125]. Bolouri et al. observed that live hAECs vaccination protected against melanoma and prolonged mouse survival effectively [126]. Subsequently, hAEC-derived small-EV (AD-sEV) was verified to exacerbate the Warburg effect, accelerate arginine depletion, and promote apoptosis in B16-F10 melanoma cells, by counteracting tumors by altering the metabolism of cancer cells. In addition, they inoculated AD-sEV pretreated B16-F10 cells into mice and observed that the pretreatment significantly prevented tumor formation and progression and prolonged survival. Notably, the protective effect against cancer was attributed to AD-sEV, not cross-reactive antibodies, as evidenced by the lack of protection with hAEC lysate vaccination or heterotopic tumor cell injection in AEC-vaccinated mice. Therefore, AD-sEV could act as an anti-cancer agent, distinct from immune-mediated tumor suppression.

### Serum-derived EVs in melanoma vaccines

Subcutaneous injection of serum exosomes loaded with different peptide neoantigens can effectively promote LN homing and DC uptake, which can significantly enhance the immunogenicity of the peptide neoantigens and trigger a potent anti-tumor immune response [127]. Zhang et al. successfully decorated peptide neoantigens M21, M27, and M33 onto serum exosomes, forming peptide neoantigen-coated exosomes (EXONeAgs) [128]. In melanoma mice, EXONeAgs increased the number of CD8+ and CD4+ T cells and activated DCs more efficiently, significantly up-regulated the DC activation marker proteins, CD80 and CD86, which further augmented the immune response, and triggered an efficacious antigen-specific immune response and tumor suppression. Therefore, serum exosomes demonstrate immense promise as delivery carriers and offer novel avenues for personalized immunotherapy leveraging peptide neoantigens.

### Bacteria-derived EVs in melanoma vaccines

Outer membrane vesicles (OMVs), a nanoscale lipid bilayer structure released by Gram-negative bacteria, possess great potential in vaccine development and drug delivery systems owing to their excellent tumor-targeting properties, membrane stability, immunogenicity, and permeability [129]. Cheng et al. incorporated the tumor antigen peptide OVA257-264 with ClyA protein on the surface of an OMV, thus effectively eliciting maturation and expression of CD80 and CD86 in bone marrow dendritic cells (BMDCs), triggering tumor antigen-specific T-cell immune responses, and markedly attenuating lung metastasis in the B16 melanoma model [130]. In addition, OMVs expressing OVA257-264 and OVA223-339 antigens together promoted more robust anti-tumor immune responses.

**Fig. 3:**
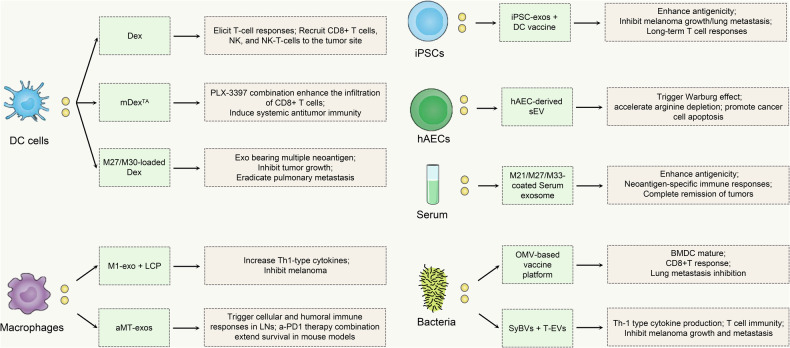
Strategies for melanoma vaccine construction based on non-melanoma-derived EVs. A variety of different sources of EVs have been validated for melanoma vaccine construction, including those derived from DC cells (poly(I:C), maturation cocktail/tumor antigen, M27/M30), Macrophages (LCP vaccine, tumor nuclei), iPSCs (iPSC-exos), hAECs (AEC-derived sEV), Serum (M21/M27/M33), Bacteria (GEM, PLGA-RA NPs). Bone marrow dendritic cells (BMDCs), cancer-associated fibroblasts (CAFs), cytotoxic T lymphocytes (CTLs), dendritic cell-derived exosomes (Dex), dendritic cells (DCs), human amniotic epithelial cells (hAECs), induced pluripotent stem cells (iPSCs), lipid calcium phosphate (LCP), myeloid-derived suppressor cells (MDSCs), outer membrane vesicles (OMVs), programmed cell death-Ligand 1 (PD-L1), synthetic bacterial vesicles (SyBVs), tumor-derived EVs (T-EVs).

Synthetic bacterial vesicles (SyBVs) were prepared via specific biochemical treatments that provoked nontoxic or pro-inflammatory responses, and activated BMDCs, inducing Th1-type cytokine production and expression of maturation markers [131]. In addition, SyBVs demonstrated superior immunoadjuvant activity and safety compared to common commercial adjuvants. The immunotherapy based on SyBVs with T-EVs significantly enhanced the infiltration of CD8+ T cells and NK cells at the tumor site, facilitated IFN-γ production, strengthened tumor-specific CTL responses, increased the levels of tumor-specific IgG associated with Th1 immune responses, and effectively inhibited the growth and metastasis of melanoma. The immunotherapy used in conjunction with anti-PD-1 inhibitors demonstrated synergistic effects, providing a novel cancer vaccine platform for combating melanoma (Fig. 3, Table 2) .

**Table 2:** Non-melanoma-derived EVs for constructing melanoma vaccines.

Name	Source of exosomes	EV Vaccine Antigens	Main function	Ref.
OVA-loaded Dex	DCs	Poly (I:C), B16-F10-OVA	Eliciting T-cell responses and recruiting cytotoxic CD8+ T cells, NK, and NK-T cells to the tumor site	[110]
mDex^TA^	Bone marrow-derived dendritic cells	Maturation cocktail,B16-F10 cell lysate antigen	Strengthening the infiltration of CD8+ T cells in TME, facilitating a shift from the Th1/Th2 balance to Th1 predominance, and decreasing TAMs and MDSCs in the B16-F10 Murine Melanoma Model, thereby inhibiting tumor growth and enhancing survival rates	[112]
M27 and M30-loaded Dex	DCs	M27 peptide, M30 peptide	Inhibiting tumor growth, extending survival periods, improving survival rates, delaying tumor progression, eradicating pulmonary metastasis in therapeutic, prophylactic, and metastatic B16-F10 melanoma models	[114]
M1-exo+LCP nanoparticle vaccine	M1 Macrophages	TRP2 peptide	Activating macrophages and DCs, thereby promoting the release of Th1-type cytokines	[119]
aMT-exos	M1-like macrophages	B16 mouse melanoma cell nuclei	Triggering cellular and humoral immune responses in LNs, increasing the immunoreactive component of TME. Extending survival in mouse models of metastatic and post-surgical tumor recurrence when combined with a-PD1 therapy	[121]
DCs pulsed with iPSC-exos vaccine	iPSCs		Inhibiting tumor growth and lung metastasis, inducing durable immune memory to prevent tumor recurrence	[123]
hAEC-derived small-EVs	hAECs		Preventing tumor formation and progression and prolonging survival	[126]
Peptide neoantigen-coated exosomes	Serum exosomes	peptide neoantigens M21, M27, and M33	Increasing the number of CD8+ and CD4+ T cells and activating DCs, significantly up-regulating the DC activation marker proteins, CD80 and CD86	[128]
OMV-based vaccine platform	*E. coli*	OVA257-264, OVA223-339, ClyA protein, TRP2	Eliciting maturation and expression of CD80 and CD86 in BMDCs, triggering tumor antigen-specific T-cell immune responses, and markedly attenuating lung metastasis in the B16 melanoma model	[130]
SyBVs	*E. coli*	SyBVs, T-EVs	Slowing melanoma tumor growth through metastatic inhibition when combined with T-EV and showing synergism with anti‐PD‐1 immunotherapy	[131]

## Discussion

Melanoma is a deadly form of skin cancer that is increasingly affecting younger populations. Currently, vaccine therapies, such as Keytruda and mRNA-based cancer vaccines (mRNA-4157/V940) have demonstrated potential in clinical trials to reduce recurrence and mortality risks, yet they are still under further investigation and refinement [132, 133]. EVs are capable of carrying and delivering specific antigens, and are promising vaccine carriers with significant potential to enhance the efficacy of anti-tumor vaccines. In this review, we have emphasized the design and functions of multiple source-derived EVs as melanoma vaccines. Several issues and challenges pertaining to the use of EVs in melanoma vaccines still need to be resolved, especially the acquirement of EVs, strategies for vaccine construction, vaccine effectiveness and safety, and clinical translation of vaccines [134].

Firstly, for the acquirement of EVs, the generation and refinement of EVs is an intricate and multifaceted process [135]. Currently, there are no universally standardized methods for their production and purification, with the principal methodologies encompassing ultracentrifugation, ultrafiltration, and size-exclusion chromatography. However, these techniques still yearn for enhancement in aspects of yield, purity, and repeatability. Furthermore, the purity, stability, and quality control of the prepared EV products present significant challenges in the preparation of EV-derived vaccines. Ensuring the purity, stability, and rigorous quality control of the resultant EV products poses substantial challenges in the development of EV-based vaccines.

In the strategic construction of EV-based melanoma vaccines, various cellular sources of EVs have been reported, including melanoma cells, immune cells, stem cells, serum, and even bacteria [136]. EVs loaded with TSAs, those emanating from antigen-presenting DCs, or EVs secreted by tumor-infiltrating macrophages, have all exhibited the ability to provoke both the adaptive and innate immune response. This immune activation is achieved through the activation of DCs, which in turn prime CD8+ T cells to unleash their cytotoxic potential, targeting and destroying melanoma cells. However, comparative analyses of EV vaccines from different origins, addressing genetic variability, bioactivity, and immune activation, have not yet been conducted. Incorporating targeting ligands, antigens, peptide adjuvants, as well as the diverse biomolecules inherently carried by EVs, is a critical strategy to enhance the immune-stimulating capacity. Nonetheless, the efficient incorporation of antigens into EVs and the integration of targeting units for precise tumor cell specificity, while minimizing the impact on normal cells, continues to be a pivotal scientific challenge [137].

EVs have demonstrated potential for treating a wide range of diseases including cancer, cardiovascular diseases, neurodegenerative disorders, diabetes, and various immune-related diseases [138]. Clinical trials are particularly important as they accurately reflect the current clinical status and level of progress of drug-based treatment strategies. After carefully reviewing, we found one clinical trial of EV-based vaccines for melanoma. In 2005, Escudie et al. reported the first clinical trial of EV-based vaccines for melanoma. This Phase I clinical trial adopted autologous Dex pulsed with MAGE 3 peptides to treat patients with stage III/IV melanoma, enrolling 15 patients who received 4 doses of the exosome vaccine [139]. No severe toxic events were observed, only mild fever and local inflammation at the injection site, which confirmed the safety of the exosome vaccines. During the treatment, one patient exhibited partial therapeutic effects with a specific T-cell response in the tumor bed and depigmentation around the naevi, accompanied by tumor shrinkage, indicating the potential efficacy of the exosome therapy. Interestingly, despite no MAGE 3-specific CD4+ and CD8+ T cell responses detected in peripheral blood, there was an increase in NK cells at tumor-infiltrated sites in 8 patients. However, the clinical benefits were limited due to the failure of the exosome vaccines to elicit a robust T-cell response.

Lastly, for clinical translation of vaccines, the safety and efficacy of EV-based melanoma vaccines warrants in-depth investigation. Concerns persist regarding the potential for EV vaccines to elicit excessive anti-tumor immune responses or to produce off-target effects that may result in toxicity to normal tissues. Additionally, it is imperative to determine the optimal dosage and administration frequency, along with potential side effects and risks. To date, EV vaccines have not been widely adopted in clinical settings for the treatment of melanoma, primarily due to the significant challenges associated with scaling up production to meet clinical demands, even after their effectiveness and safety have been demonstrated under laboratory conditions. Scale-up production confronts issues such as enhancing manufacturing efficiency, reducing costs, and ensuring product quality and consistency. Moreover, the therapeutic efficacy of EVs in tumor vaccines requires validation through extensive clinical trials, necessitating the enrollment of a specific number of patients, adequate long-term follow-up, identification of appropriate endpoints, as well as thorough data analysis and interpretation.

## Conclusion

Collectively, here we have concluded the reported melanoma vaccines constructed from multiple sources of EVs, including melanoma cells, DCs, macrophages, iPSCs, hAECs, serum, engineered nanos, and bacteria. The established EV melanoma vaccines, by virtue of antigen expression and integration of targeting units, have demonstrated robust anti-tumor immune activation effects through the stimulation of DCs and CD8+ T cells. Therefore, the innovative strategies of EV melanoma vaccines are promising in melanoma vaccine development.
